# Value of Dual-Energy CT Perfusion Analysis in Patients with Acute Pancreatitis: Correlation and Discriminative Diagnostic Accuracy with Varying Disease Severity

**DOI:** 10.3390/diagnostics12112601

**Published:** 2022-10-27

**Authors:** Scherwin Mahmoudi, Simon Martin, Vitali Koch, Leon David Gruenewald, Simon Bernatz, Tommaso D’Angelo, Thomas J. Vogl, Christian Booz, Ibrahim Yel

**Affiliations:** 1 Department of Diagnostic and Interventional Radiology, University Hospital Frankfurt, 60590 Frankfurt, Germany; 2 Department of Biomedical Sciences and Morphological and Functional Imaging, University Hospital Messina, 98100 Messina, Italy; 3 Department of Radiology and Nuclear Medicine, Erasmus MC, 3015 Rotterdam, The Netherlands

**Keywords:** DECT, computed tomography, iodine quantification, iodine density, material density, acute pancreatitis, modified CT severity index

## Abstract

Background: This study investigates the correlation and discriminative diagnostic accuracy of dual-energy CT (DECT)-derived imaging biomarkers in patients with acute pancreatitis of varying severity. Methods: In this retrospective study, we included 51 patients with acute pancreatitis who had undergone portal-venous phase DECT of the abdomen. Three blinded readers independently performed region-of-interest measurements on DECT images in the inflammatory pancreatic parenchyma. The correlation between modified CT severity index (CTSI) and quantitative imaging parameters was investigated using Pearson correlation coefficient. We performed receiver operator curve (ROC) analysis to assess diagnostic accuracy of the quantitative image parameters for the differentiation between mild/moderate versus severe acute pancreatitis. The optimal discriminative cut-off value to diagnose severe acute pancreatitis was determined using the Youden index. Results: Moderate correlations were found between CTSI scores and iodine density (Pearson’s correlation coefficient r = −0.65; *p* < 0.001), as well as attenuation (r = −0.55; *p* < 0.001) and normalized iodine uptake (r = −0.50; *p* < 0.001). ROC curve analysis revealed highest ability to differentiate mild/moderate from severe acute pancreatitis for iodine density (AUC = 0.86, 95% confidence interval 0.75 to 0.97). An optimal iodine density threshold of ≤1.63 mg/mL was found to indicate severe acute pancreatitis with a sensitivity of 81.3% and specificity of 77.1%. Conclusion: DECT-derived iodine density correlates with acute pancreatitis severity and may facilitate prediction of severe acute pancreatitis.

## 1. Introduction

Acute pancreatitis is a sudden, primarily non-infectious inflammation caused by the enzymatic self-digestion of the pancreas [1]. Most episodes of acute pancreatitis are mild and self-limiting. However, severe acute pancreatitis occurs in 15–20% of patients and has a higher risk of morbidity and mortality [2]. Therefore, early diagnosis with the optimal imaging modality and risk stratification is essential for prognosis [3]. Whereas ultrasound represents the first-line imaging modality for confirming the diagnosis of acute pancreatitis [4], computed tomography (CT) is used to assess the extent, course, and complications of acute pancreatitis. CT allows differentiation between peripancreatic fluid collection and necrosis, as well as the evaluation of the size and extent of pseudocysts or wall-forming necrosis [5].

Several scores aim to predict the severity of acute pancreatitis [2,6]. The modified CT severity index (CTSI) was established for CT-based evaluation to classify acute pancreatitis as mild, moderate, and severe [7]. The CTSI findings have been found to correlate well with clinical indices of severity. However, interobserver variability may occur and can result in different scores for the same patient [8].

Dual-energy CT (DECT), introduced as a first-generation dual-source CT in 2006, provides a wide range of post-processing techniques and allows for a wide range of image series aside from conventional monochromatic images [9]. The DECT material decomposition algorithms can provide additional information about the distribution and concentration of a specific material (e.g., iodine, fat, calcium) within tissues [10]. The clinical applications of DECT post-processing material decomposition, particularly iodine quantification and fat fraction, have been extensively investigated in gastrointestinal imaging over recent years [11]. In more detail, DECT iodine quantification has shown promising results in the diagnosis of acute pancreatitis, even in the early stages when typical findings are not apparent on standard CT images [12,13]. To date, only one recently published study evaluates the correlation of iodine uptake in rapid-kV switching DECT images with acute pancreatitis of varying severity [14]. However, the classification of acute pancreatitis severity following iodine quantification in dual-source DECT is missing so far. The aim of our study was to investigate the correlation and discriminative diagnostic accuracy of dual-source DECT iodine uptake and fat fraction analysis in patients with acute pancreatitis of varying severity.

## 2. Materials and Methods

The ethical review board of our institution approved this retrospective study and waived written informed consent. The study was conducted according to the guidelines of the Declaration of Helsinki.

### 2.1. Study Population

In this retrospective, single-center study, we reviewed our database for patients with clinically confirmed acute pancreatitis and included a total of 51 consecutive patients with clinically confirmed acute pancreatitis who had undergone contrast-enhanced DECT scans between 08/16 and 01/19 in portal-venous phase. Clinical diagnosis of acute pancreatitis was made when two of the three following criteria were met: (I) characteristic abdominal pain, (II) elevated serum pancreatic enzymes: lipase or amylase ≥ 3 × upper limit of normal, (III) characteristic findings of acute pancreatitis on cross-sectional imaging [15,16].

**Figure 1:**
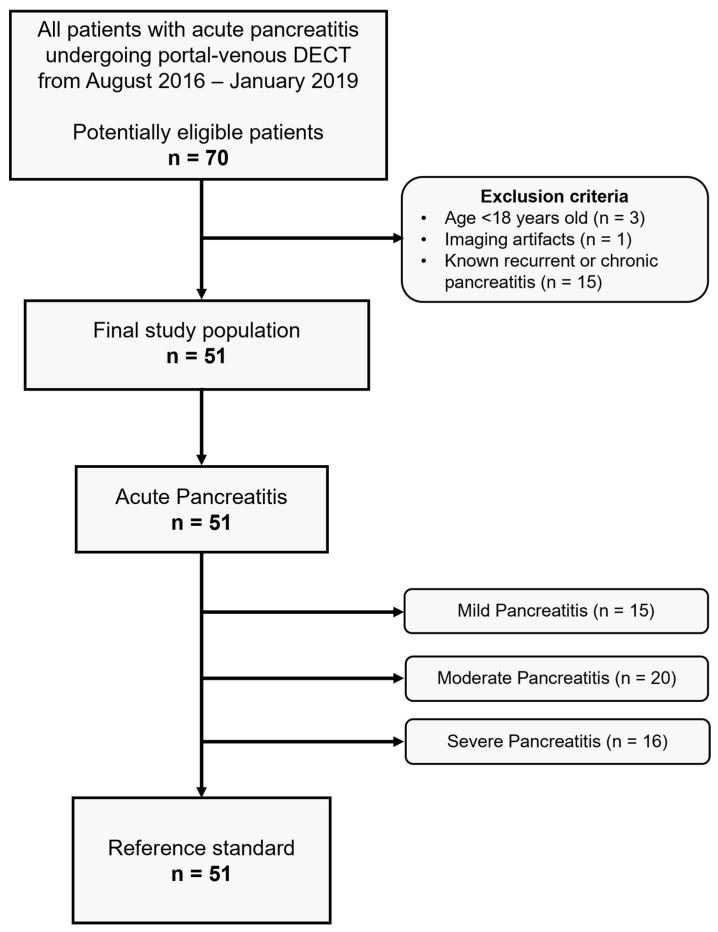
STARD flowchart of study inclusion.

Exclusion criteria were (I) age <18 years old, (II) imaging artifacts, (III) known recurrent or chronic pancreatitis. In cases of patients with multiple CTs, only the first DECT was included. Clinical data (date of birth, gender, tumor stage, tumor size, laboratory parameters, and follow-up) were collected from electronic medical records. All data were obtained in clinical routine. Figure 1 depicts the flowchart of patient inclusion according to Standards for Reporting Diagnostic Accuracy Studies (STARD).

### 2.2. CT Acquisition Protocol and Image Reconstruction

All CT scans were performed on the same third-generation dual-source DECT (Somatom Force; Siemens Healthineers, Forchheim, Germany) with the following default settings of the x-ray tubes: tube A: 100 kV, 190 mAs; tube B: 150 kV, 95 mAs; additional tin filter (Selective Photon Shield II, Siemens Healthineers, Forchheim, Germany). Image acquisition was performed in craniocaudal direction during inspiratory breath-hold. Rotation time was 0.5 s. Collimation was 2 × 192 × 0.6 mm. The applied protocol contained automatic attenuation-based tube current modulation (CARE Dose 4D; Siemens Healthineers, Forchheim, Germany).

A non-ionic contrast agent at a dose of 1.2 mL/kg of body weight with a maximum of 120 mL was injected through a peripheral vein of the forearm. Contrast media administration was performed with a flow of 2–3 mL/s with a maximum of 120 mL, followed by an 80 mL saline flush. Image acquisition during venous phase of contrast enhancement started 70 s after contrast agent injection. An iterative reconstruction algorithm (ADMIRE^®^, Siemens Healthineers, Forchheim, Germany) was used for image reconstruction. CT dose index (CTDI) and dose-length-product (DLP) were recorded from the patient protocol.

### 2.3. DECT Image Postprocessing and Image Analysis

A 3D multi-modality workstation (syngo.via, version VB10B, Siemens Healthineers, Forchheim, Germany) with an iodine subtraction algorithm (Liver VNC, Siemens Healthineers, Forchheim, Germany) was used for DECT material decomposition image reconstruction.

**Figure 2:**
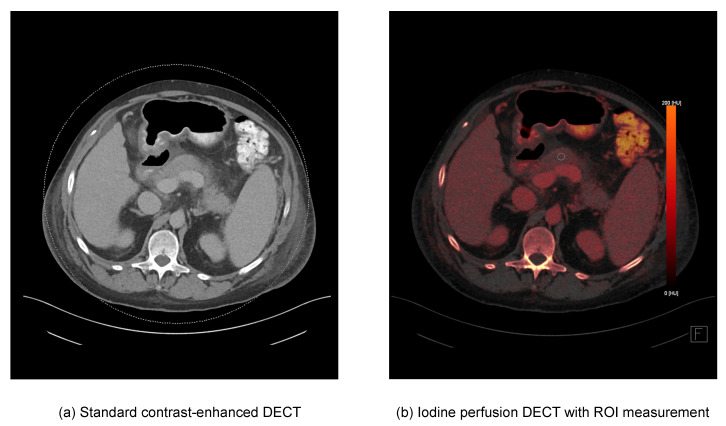
DECT iodine quantification segmentation. Axial DECT images of a 56-year-old male patient with moderate acute pancreatitis (CTSI = 6). (**a**) Standard contrast-enhanced DECT, (**b**) iodine perfusion DECT with ROI measurement for DECT material decomposition analysis.

Three independent radiologists with different levels of experience (I, 2 years of experience, II, 3 years of experience, III, 6 years of experience) performed region of interest (ROI) measurements of the center of the pancreatic corpus on DECT iodine perfusion images (Figure 2). In total, three segmentations per patient were performed.

ROI measurements for DECT material decomposition analysis were drawn with a diameter of 1.0 cm, sparing surrounding structures, vessels, pancreatic calcifications, and pancreatic necrosis. In addition, one ROI measurement per patient was performed in the abdominal aorta at the level of the celiac trunk by the most experienced radiologist (III). Attenuation values and DECT material decomposition values, including iodine density and fat fraction of the pancreas, were calculated.

Normalized iodine uptake was calculated using the following formula:

(1) $$Normalized\;iodine\;uptake=\frac{Iodine\;Density_{\;lesion}}{Iodine\;Density_{\;aorta}}$$

The mean value of the three measurements was used for further analysis.

All three radiologists were blinded to the clinical records and CT reports.

### 2.4. Statistical Analysis

Statistical analysis was performed using Stata (Version 13, StataCorp, College Station, TX, USA). Numeric values of continuous variables were reported as mean ± standard deviation. Categorial variables were expressed as percentages. To analyze data regarding normal distribution, the Kolmogorov–Smirnov test was used. We used an analysis of variance (ANOVA) test for data sowing continuous distribution. Data showing non-normal distribution were analyzed with Wilcoxon Signed-Ranked test.

All cases were evaluated based on the modified CTSI, taking into account pancreatic inflammation, necrosis, and extrapancreatic complications [17]. According to the modified CTSI, severity of acute pancreatitis was divided into mild, moderate, and severe. CTSI scoring was performed on grayscale CT images.

Intraclass-Correlation Coefficient (ICC) was used in a two-way mixed-effects model to calculate interobserver agreement among the three radiologists. ICC was interpreted according to Koo/Li [18]: ICC < 0.50 = poor agreement, ICC 0.50–0.75 = moderate agreement, ICC 0.75–0.90 = good agreement, and ICC > 0.9 = excellent agreement.

Mean values of attenuation values, iodine density, fat fraction, and normalized iodine uptake were compared between mild, moderate, and severe acute pancreatitis. The correlation between disease severity and the quantitative imaging parameters was investigated using Pearson correlation coefficient.

Methods pertaining to the assessment of model performance refer to a univariate logistic regression model for the outcome of mild/moderate versus severe acute pancreatitis, including the continuous quantitative image parameters as independent variables. The discriminative ability of the model was assessed by plotting the receiver operating characteristic (ROC) curve and calculating the area under the curve (AUC).

We established threshold values for the best quantitative image parameter to differentiate between mild/moderate and severe acute pancreatitis using the Youden index.

A *p*-value (*p*) ≤ 0.05 indicated statistical significance.

## 3. Results

We included a total of 51 consecutive patients (male, 39; median age 54.0 (interquartile range (IQR) 41.4–65.6). The mean modified CTSI within all included patients with acute pancreatitis was 5.2 ± 3.0. DECT radiation metrics in venous phase acquisition were 10.2 ± 4.3 mGy for mean volume CTDI and 561.7 ± 278.2 mGy × cm for mean DLP. Baseline patient and clinical characteristics are summarized in Table 1.

Mean attenuation values of inflammatory pancreatic parenchyma significantly differed between mild acute pancreatitis (90.0 ± 12.9), moderate acute pancreatitis (76.0 ± 13.8), and severe pancreatitis (63.3 ± 17.7) (*p* < 0.001).

Mean values of iodine density and normalized iodine uptake significantly differed between mild acute pancreatitis (2.3 ± 0.5 mg/mL; 0.52 ± 0.13), moderate acute pancreatitis (1.8 ± 0.4 mg/mL; 0.4 ± 0.1), and severe acute pancreatitis (1.4 ± 0.4 mg/mL; 0.4 ± 0.1) (*p* < 0.001; *p* = 0.002).

No significant differences were found for fat fraction analysis between mild acute pancreatitis (11.2 ± 7.2%), moderate acute pancreatitis (13.9 ± 6.8%), and severe acute pancreatitis (16.0 ± 8.4%) (*p* = 0.221). The results of quantitative image parameter analyses are displayed in Table 2. Importantly, ICC revealed excellent reliability for iodine density (0.91) and normalized iodine uptake (0.93), and good reliability for attenuation (0.90) and fat fraction (0.81) between the three independent radiologists.

Moderate correlation was found between CTSI scores and mean iodine density (Pearson’s correlation coefficient r = −0.6483; *p* < 0.001) [19]. Moderate correlations with CTSI scores were also determined for mean attenuation (Pearson’s correlation coefficient r = −0.5856; *p* < 0.001) and normalized iodine uptake (Pearson’s correlation coefficient r = −0.5009; *p* < 0.001) [19] with lower correlation compared to iodine density. No significant correlation was reported for fat fraction.

**Figure 3:**
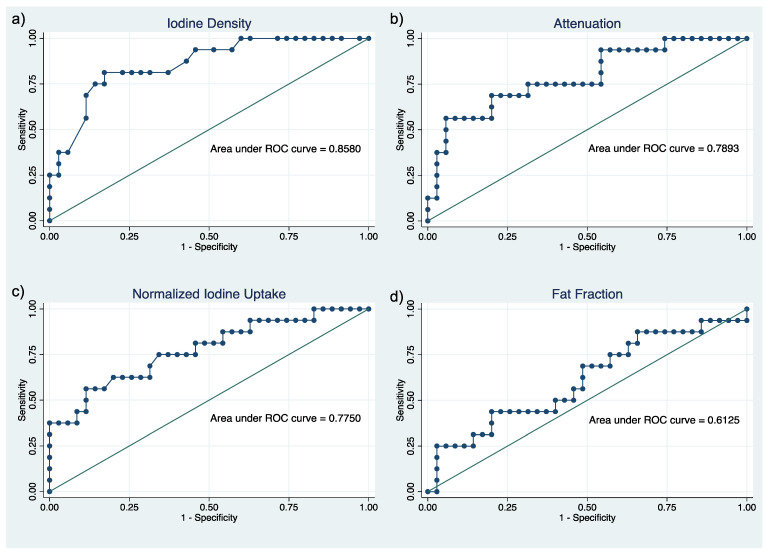
Receiver operating characteristic (ROC) curve analysis. Receiver operating characteristic (ROC) curve analysis for different quantitative image parameters. (**a**) Iodine density, (**b**) attenuation, (**c**) normalized iodine uptake, (**d**) fat fraction.

ROC curve analysis revealed the highest ability to differentiate mild/moderate acute pancreatitis from severe acute pancreatitis for iodine density (AUC = 0.86, 95 % confidence interval [CI] 0.75 to 0.97; Figure 3a). Lower discriminative ability was observed for attenuation (AUC = 0.79; CI, 0.65 to 0.93; Figure 3b), normalized iodine uptake (AUC = 0.78; CI, 0.63 to 0.92; Figure 3c) and fat fraction (AUC = 0.61; CI, 0.44 to 0.79; Figure 3d). Correlation with acute pancreatitis severity, as well as results of diagnostic performance of the quantitative image parameters to differentiate between mild/moderate and severe acute pancreatitis are summarized in Table 3.

An iodine density threshold of ≤1.63 mg/mL was found to indicate a severe acute pancreatitis with a sensitivity of 81.3% and specificity of 77.1%.

## 4. Discussion

Contrast-enhanced CT is an important imaging modality for the assessment of extent, course, and complications of acute pancreatitis [5]. The aim of this study was to evaluate the potential of quantitative parameters derived from contrast-enhanced DECT to differentiate acute pancreatitis of varying disease severity. To our knowledge, this is the first study that investigates this topic in a dual-source DECT.

Our data suggest that DECT-derived quantitative image parameters allow for the prediction of acute pancreatitis severity. To be more precise, we were able to identify iodine density as a reliable imaging biomarker that allows the prediction of acute pancreatitis severity.

Notably, iodine density yielded good diagnostic performance, indicating that the iodine density-based model profoundly correlated with varying severity of acute pancreatitis. The high performance was confirmed by the Youden Index, defining an optimal threshold of 1.63 mg/dL to discriminate between mild/moderate and severe acute pancreatitis with a sensitivity of 81.3% and specificity of 77.1%.

In recent years, the application of DECT post-processing techniques has strongly emerged in gastrointestinal imaging [20,21]. Several studies identified DECT post-processing techniques as a reliable tool for detecting and assessing pancreas-related diseases, including pancreatic carcinoma and pancreatitis [22]. In a study from 2015, the authors demonstrated improved image quality of DECT-derived noise reduction algorithms that allow improved lesion delineation in patients with pancreatic carcinoma [23]. In a recently published study, Mathy et al. investigated the value of DECT-derived imaging biomarkers for the detection of local pancreatic carcinoma recurrence after surgical resection [24]. In their study from 2021, the authors were able to identify higher iodine concentrations in malignant pancreatic parenchyma compared to non-specific pancreatic postoperative soft tissue. By applying different X-ray spectra in DECT, iodine concentration can be measured quantitatively to reflect information about vascular supply of tumors and, therefore, may be increased in cases of pancreatic carcinoma [25]. In contrast to pancreatic malignancies, our study could demonstrate lower iodine concentrations of pancreatic tissue in severely inflammatory affected areas. These findings may be explained by the higher probability of necrotic areas in severe cases of acute pancreatitis, which correlates with low perfused parenchymatous tissue and results in lower iodine concentration. Additionally, the increased capillary permeability with subsequent fluid loss in inflammatory affected pancreatic parenchyma may contribute to lower iodine concentrations in cases of acute pancreatitis [26]. Similar findings were presented by Martin et al. in 2018 [13]. In their retrospective study, the authors proposed an iodine density cut-off of ≤2.1 mg/dL to diagnose acute pancreatitis in contrast-enhanced CT in early stages. Our results further approve these findings by confirming low iodine concentrations in inflammatory pancreatic parenchyma. Additionally, an additional cut-off to differentiate mild/moderate from severe pancreatitis might be of clinical relevance since patients who suffer from severe acute pancreatitis have a higher risk of morbidity and mortality [2]. Especially in early cases of acute pancreatitis, when radiographic CT features may be variable and difficult to detect based on subjective evaluation, iodine quantification may be helpful to detect early cases of patients with severe acute pancreatitis [27,28], facilitating early treatment and prognosis. Additionally, the use of DECT-derived iodine density in patients with acute pancreatitis may add relevant clinical information in equivocal cases compared to standard subjective image evaluation.

Our study has several limitations, which have to be taken into account.

We performed a retrospective single-center study. As a consequence, our sample size is modest and may lead to case selection bias. Additionally, our study included 51 patients, and a larger cohort might have been favorable. This might reduce the generalizability of our findings. However, the case number of our cohort is similar to comparable studies that investigate CT-based evaluation of pancreatitis and is limited due to the role of ultrasound as the first-line imaging modality for the confirmation of the diagnosis of acute pancreatitis. Another limitation is that we solely investigated portal-venous phase CT scans. However, the choice of a different phase scan, as, e.g., reported by Martin et al. in 2018 for pancreatic-phase images, showed comparable results [13]. Even though the radiologists were briefed to place the ROI measurement in the central region of the pancreas corpus to maintain comparability, results may vary depending on the site and number of ROI measurements.

Last, our institute works with the dual-source DECT system. The findings of our study may restrict the application of iodine quantification to dual-source DECT systems, although several studies demonstrated comparable iodine density accuracies for the different DECT systems, including dual-source DECT, dual-layer DECT, and rapid-kV switching DECT [29,30]. Further prospective studies should aim to validate thresholds suggested by our data, particularly to establish the use of iodine density to detect severe acute pancreatitis at early stage.

In conclusion, DECT-derived quantitative image parameters allow for the prediction of acute pancreatitis severity. First-pass perfusion analysis in contrast-enhanced DECT shows high diagnostic accuracy in diagnosing severe acute pancreatitis. We identified iodine density as a reliable imaging biomarker that correlates with CTSI score and allows the prediction of acute pancreatitis severity.

## Figures and Tables

**Table 1:** Patient characteristics.

Parameters	Value
Number of patients (n)	51
Male / Female (n)	39/12
Age at date of CT scan (median years, range)	54.0 (19.4–92.6)
Mean CTDI (mGy)	10.2 ± 4.3 (3.5–26.1)
Mean DLP (mGy × cm)	561.7 ± 278.2 (210.5–1687.5)
Lipase (U/L)	2217.3 ± 3614.6 (7–14137)
Mean Modified CTSI	5.2 ± 3.0
Modified CTSI (n)	
Mild	15
Moderate	20
Severe	16

If not depicted otherwise, the numbers without parenthesis depict absolute numbers. Data in round parenthesis are the min/max values. Continuous variables are shown as mean with standard deviation. Abbreviations: CT, computed tomography; CTDI, computed tomography dose index; CTSI, computed tomography severity index; DLP, dose-length product.

**Table 2:** Comparison of quantitative image parameters.

Parameters	Mild Acute Pancreatitis	Moderate Acute Pancreatitis	Severe Acute Pancreatitis	*p*-Value
Attenuation (HU)	89.97 ± 12.93	76.00 ± 13.80	63.31 ± 17.66	<0.001
Iodine density (mg/mL)	2.28 ± 0.51	1.84 ± 0.43	1.39 ± 0.35	<0.001
Normalized iodine uptake	0.52 ± 0.13	0.44 ± 0.14	0.35 ± 0.11	0.002
Fat fraction (%)	11.24 ± 7.20	13.88 ± 6.82	15.95 ± 8.37	0.221

Comparison of attenuation, iodine density, normalized iodine uptake and fat fraction mean scores ± standard deviation between varying disease severity of pancreatitis using analysis of variance (ANOVA) test. Abbreviations: HU (Hounsfield Units).

**Table 3:** Correlation with acute pancreatitis severity and diagnostic performance to discriminate between mild/moderate acute pancreatitis and severe acute pancreatitis.

Parameters	Pearson Correlation	AUC (95% CI)	ICC (95% CI)
Iodine density	−0.6483; *p* < 0.001	0.86 (0.75–0.97)	0.91 (0.86–0.95)
Attenuation	−0.5856; *p* < 0.001	0.79 (0.65–0.93)	0.90 (0.84–0.94)
Normalized iodine uptake	−0.5009; *p* < 0.001	0.78 (0.63–0.92)	0.93 (0.89–0.96)
Fat fraction	+0.2416; *p* = 0.0876	0.61 (0.44–0.79)	0.81 (0.69–0.88)

Correlation with acute pancreatitis severity and diagnostic performance to discriminate between mild/moderate acute pancreatitis and severe acute pancreatitis. Abbreviations: AUC (area under the curve), CI (confidence interval), ICC (Intraclass correlation coefficient).

## Data Availability

The data presented in this study are available on request from the corresponding author. The data are not publicly available due to data protection.

## Abbreviations

AUC	Area under the curve
CT	Computed tomography
CTDI	Computed tomography dose index
CTSI	Computed tomography severity index
DECT	Dual-energy computed tomography
DLP	Dose-length product
HU	Hounsfield Units
ICC	Intraclass correlation coefficient
ROC	Receiver operating characteristic
ROI	Region of interest
STARD	Standards for Reporting Diagnostic Accuracy Studies
